# Medicines as Common Commodities or Powerful Potions? What Makes Medicines Reusable in People’s Eyes

**DOI:** 10.3390/pharmacy9020088

**Published:** 2021-04-20

**Authors:** Monica Chauhan, Hamza Alhamad, Rachel McCrindle, Terence K. L. Hui, R. Simon Sherratt, Parastou Donyai

**Affiliations:** 1 Reading School of Pharmacy, University of Reading, Reading RG6 6AP, UK; monicachauhan15@hotmail.co.uk (M.C.); halhamad@zu.edu.jo (H.A.); 2 Department of Pharmacy, Zarqa University, Zarqa 132222, Jordan; 3 Department of Biomedical Engineering, School of Biological Sciences, University of Reading, Reading RG6 6AY, UK; r.j.mccrindle@reading.ac.uk (R.M.); t.hui@reading.ac.uk (T.K.L.H.); r.s.sherratt@reading.ac.uk (R.S.S.)

**Keywords:** medicines, reuse, recycle, medicines reuse, attitudes

## Abstract

Background: Medicines reuse involves dispensing quality-checked, unused medication returned by one patient for another, instead of disposal as waste. This is prohibited in UK community pharmacy because storage conditions in a patient’s home could potentially impact on the quality, safety and efficacy of returned medicines. Our 2017 survey examining patients’ intentions to reuse medicines found many favoured medicines reuse. Our aim was to analyse the qualitative comments to explore people’s interpretations of what makes medicines (non-)reusable. Methods: Thematic analysis was used to scrutinize 210 valid qualitative responses to the survey to delineate the themes and super-ordinate categories. Results: Two categories were “medicines as common commodities” versus “medicines as powerful potions”. People’s ideas about medicines aligned closely with other common commodities, exchanged from manufacturers to consumers, with many seeing medicines as commercial goods with economic value sanctioning their reuse. Fewer of the comments aligned with the biomedical notion of medicines as powerful potions, regulated and with legal and ethical boundaries limiting their (re)use. Conclusion: People’s pro-medicines-reuse beliefs align with perceptions of medicines as common commodities. This helps explain why patients returning their medicines to community pharmacies want these to be recycled. It could also explain why governments permit medicines reuse in emergencies.

## 1. Introduction

Medicines reuse is the idea that quality-checked, unused, prescribed medication returned by one patient can be re-dispensed for another patient instead of disposal as waste. Medicines reuse is currently prohibited in the UK community pharmacy context, mainly because the storage conditions in a patient’s home could potentially impact on the quality, safety and efficacy of returned medicines kept there, outside of the formal supply chain [1,2]. However, disregarding medicines reuse is not a sustainable position either. Firstly, a third of the cost of prescribed medicinal waste relates to medicines returned to community pharmacies for disposal [3], a problem which could arguably be addressed with the implementation of a safe medicines reuse programme. Secondly, unpredictable events such as pandemics [4] and drug shortages [5] continue to force the UK government to temporarily relax its rules on medicines reuse in any case, a situation which could be made safer with better investment and research into secure medicines reuse practices.

Internationally, doctors, academics and officials have been debating medicines reuse for many decades. Canadian doctors, for example, have called for the recycling of expensive cancer drugs for disadvantaged patients [6], and in the UK in 2012, even the then director of the NHS Sustainable Development Unit argued for research-informed debate on medicines reuse [7]. Indian academics have called for medicines reuse to be explored [8], and researchers from Italy have examined the pros and cons of donating returned medicines to organizations in Europe, Africa and Latin America against WHO’s formal advice to withhold such donations [9]. However, it is also worth noting that research shows that underground medication exchange activities are already taking place among patients, for example with diabetes medication [10], which negates these “intellectual” arguments about medicines reuse. Another example is a study in Iran, reporting the frequent self-reuse of antibiotics by people who keep their medicines in places such as their fridge in case they are needed at a later time [11]. This type of “illicit” medicines reuse practice even extends to the scavenging and the onward recycling of medicines from waste disposal sites in some developing countries [12]. Thus, the concept of medicines reuse does not just remain relevant conceptually, it can also be considered an urgent public health issue because informally it already takes place, further warranting research.

The uncertainties about the quality, safety and efficacy of returned medicines relate to the chemical and physical properties of medicines, which can be affected by fluctuations in the environment in which medicines are kept in a patient’s home, including changes in temperature, light, humidity, cleanliness and motion/agitation. It is consequently possible for the active ingredient of the medication to degrade, or the formulation to break down so that ultimately less of the medicine is available to treat the disease. Yet, some countries around the world have already instigated medicines reuse schemes but without sophisticated ways of checking for the potential impact of the storage conditions on the stability of the medication. For instance, in Athens, Greece, the GivMed programme allows people access to leftover medicines [13]. Similarly, in many states of the United States (US), medicines donation and reuse programmes exist to support those unable to afford medicines [14]. The practice also appears to have been taking place in the Kingdom of Brunei since 2006 [15]. In these schemes, donated medicines are checked by licensed pharmacists against specific criteria to allow their re-dispensing. However, these checks are largely visual and although they might prevent the re-entry of obviously damaged medicines into the system, they cannot realistically safeguard against physically or chemically degraded content being inadvertently accepted for reuse. This is because visual checks are a mere proxy marker of quality—they do not reveal the storage history of returned medicines nor the impact of that history on the contents within. Thus, for example, a product that requires cold storage could be brought back for reuse, having been kept at room temperature, without necessarily showing physical signs of damage. This is against a backdrop of research that shows, for example 58.3% of patients store their thermolabile medicines outside of the correct temperature recommendations [16].

In the UK, the COVID-19 pandemic caused the government to permit care homes and hospices to draw up standard operating procedures to enable medicines reuse if impacted by shortages [17]. However, here again, the quality checks relied on the visual inspection of any potential medicines rather than any in-depth safeguards, very similar to the protocol for the evaluation and redistribution of donated medicines used in a pilot medication recycling project in Singapore [18]. This type of practice exposes potential contradictions in the very conceptualization of returned medicines by those in positions of power—on the one hand, these are deemed potentially unsafe and must not be reused because their content might have degraded, and on the other, they are deemed safe on passing visual and expiry checks (as proxies for the potency of the active ingredient and formulated medicine inside).

When the public are asked about medicines reuse in formal studies, conflicting ideas about medicines are again highlighted. For example, people interviewed in an Australian study about medication waste questioned whether expired medicines are really totally worthless or could be somehow reused, while also referring to these as “cast-offs” [19]. In the UK too, the people we interviewed in 2016 juxtaposed the potential economic and environmental benefits of medicines reuse with stability and safety worries [20]. The latter was also a predominant feature of interviews conducted in the Netherlands in 2014/15, where the potential to prevent medication waste was set against a guarantee of product quality for any re-dispensed medication [21]. In 2017, we developed and validated a theory of planned behaviour-based medicines reuse questionnaire and used this to survey over a thousand people with at least one chronic health condition in the UK [22]. We showed that people could be encouraged to embrace medicines reuse via practical measures that illustrate the safety and quality assurance of reissued medicines, educational interventions that bolster beliefs about the pro-environmental benefits, and norm-based interventions, encouraging doctors and pharmacists to endorse the practice. Based on ours and others’ work, it is certainly clear then that ordinary people, when questioned, also recognize the need for medicines to be quality-assured if they are to be reused. What remains unresolved, however, is whether people understand the nuanced way in which the quality of medication might degrade and, in turn, need to be assured, i.e., whether they recognize medicines as complicated entities worthy of a greater level of scrutiny than visual inspections alone if their safety and quality is to be checked. This is important for the success of wide-scale medicines reuse programmes which would rely on patient uptake. The topic is also important to help explain how medicines on the one hand are deemed potentially unsafe and not reusable by policy makers (because their content might have degraded), yet on the other, deemed safe on passing visual and expiry checks in emergencies and other cases. The core interest of this paper, therefore, is to study how people conceptualize medicines and the properties that make them reusable or not.

The specific aim was to thematically analyse the qualitative responses in our 2017 survey on medicines reuse [22] to explore people’s interpretations of the properties of medicines that made them (non-)reusable in order to explore and study the presence of contradictions or conflicting ideas which both make medicines “reusable” and do not, in our participants’ view.

## 2. Materials and Methods

The primary data for this study came from our 2017 survey that employed the medicines reuse questionnaire and was completed by 1003 people who had at least one chronic health condition [22]. It is important to highlight that a quantitative analysis of the survey responses has already been published elsewhere [22]. This survey itself was developed as part of the Ph.D. of one of the co-authors (H.A.) and the publication referenced above contains full details of the questionnaire items, their development and validation, the distribution of the survey as well as the demographics of the participants [22]. The survey had a representative number of participants from across the UK in terms of gender, ethnicity, geographical location and educational level and readers are again referred to the existing publication for the participant details [22].

Within the responses, there were 210 valid qualitative comments to analyse in response to the question “If you have any comments, or ideas regarding the concept of medication reuse, please share them here”. These comments were extracted into an Excel spreadsheet for the analysis.

Thematic analysis was employed for the analysis [23]. This approach was used because it provided a way of organising the qualitative data in the form of themes: recurrent topics, ideas or statements identified across the corpus of data. P.D. reviewed all the qualitative comments to confirm that names or other information that might identify the participants had been removed. The comments were analysed manually by M.C. in consultation with P.D., according to the six phases described by Braun and Clarke [23]. The process involved familiarisation with the data, coding, searching for themes, reviewing themes, defining and naming themes, and writing up, as follows.

After familiarisation with the data, M.C. coded each comment and assigned initial “code names”. These codes first reflected what made medicines “reusable” and what did not. Consider the following three examples:

**Example** **1.** *“It is worth thinking about to save the NHS money...”*

**Example** **2.** *“If they are sealed and none taken out the pack this would help the NHS save money.”*

**Example** **3.** *“Providing the products were in date, quality checked, safety checked, and original packing I would have no objections as it should save the NHS a huge amount of money.”*

Example 1 was initially assigned the code “NHS saving” based on the essence of what was being communicated. Example 2 was also coded “NHS saving” but also with the codes “appearance” and ”packaging”. Finally, example 3 was given numerous codes, “quality”, “expiry”, “safety”, “packaging”, and “NHS savings”. This process was completed for the entire list of 210 comments. This constituted what is known as first-order coding, the lowest level of coding where the aim is simply to organize and categorize the data by capturing chunks of ideas and giving them labels in a purely descriptive way with minimal interpretation.

Once all the initial codes had been generated, it was possible to group the codes according to recurrent topics or ideas by seeing the patterns in ideas from one quote to another. This second-level coding aimed to go beyond the simple description of the data to instead interpret the meaning of the words. Here, labels were devised which captured the meaning of larger segments of the data, thus reducing the number of codes by sorting ideas into broader and more encompassing categories. Thus, for example, the initial codes of “packaging”, “expiry”, and “appearance” were grouped according to the theme of “physical appearance”. Here, the interpretive element is the description given to the category, that “the external features and overall physical appearance of a commodity are adequate to indicate what is held within. Therefore, intact sealed packaging of medicines suggests an authentic product of good quality inside”.

The final stage of coding involved drawing out the overarching themes within the data. The aim of this third-order coding was to identify superordinate constructs that were more global so that larger-scale patterns could be identified. This was completed by continuing to check and compare the ideas, checking other relevant literature in the field and even standing back from the data so that more general concepts and patterns could be drawn out. Thus, for example, the theme of “physical appearance”, identified above, was placed within the superordinate category of “medicines as common commodities” which encapsulates commonly held ideas about what makes medicines the same as any other commodity and therefore suitable for reuse. It was at this stage that the two superordinate categories, described more fully in the Results section below, were formed.

## 3. Results

Two super-ordinate categories encapsulated people’s ideas about what made medicines “reusable” or not, each with four distinct themes. The categories and themes and their explanations are provided in Table 1 and further described in the text below.

**Figure 1:**
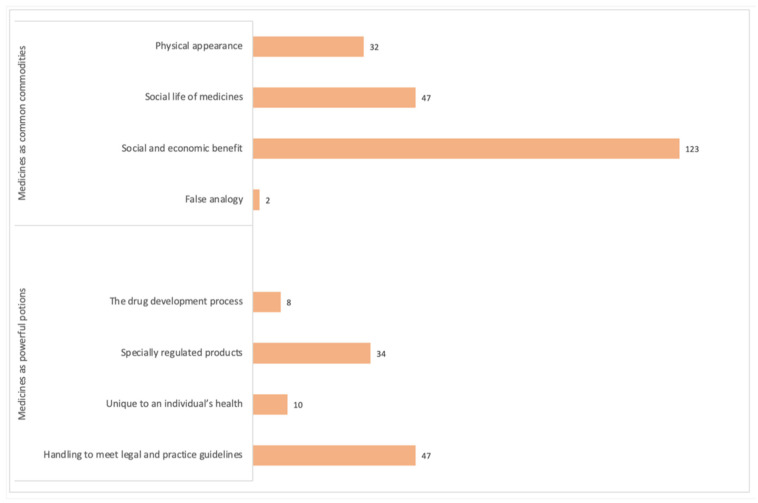
The number of times each theme was identified in the qualitative comments from the 210 survey participants. Note. Some comments were categorized according to two or more themes.

The majority of people’s views related to ideas and concepts that defined medicines as common commodities, sanctioning their reuse (Figure 1).

### 3.1. Medicines as Common Commodities

The four themes within this category relate to how people see medicines as similar to any common commodity. Most of the patient comments fell within these themes.

#### 3.1.1. Physical Appearance

This theme encapsulates the idea that the external appearance of medicines, the packaging, neatness and overall visual state, are a strong indicator of the quality of what is held within. These superficial features thus, apparently, reflect the quality and function of the drug. Individuals made positive comments about the idea of reuse by relating to different physical features of the packaging as an indicator of quality. For example, the seal on the packaging was mentioned numerous times with the idea being that a sealed product would be suitable for reuse. The logic is that a blister pack that is presented with a seal and is completely labelled with no damage such as creases or torn edges would suggest the medicine inside is unchanged, safe, and appropriate to be reused. For example:

“*When will the reuse of medication become legal? As long as it’s sealed, I would be happy*.”

(Participant 91).

“*As long as medication is in sealed blister packs showing expiry date then it has to be a good thing*.”

(Participant 23).

“*No reason at all not to re-use medication that is sealed and labelled*.”

(Participant 185).

#### 3.1.2. Social Life of Medicines

This theme originates from studies within the field of medical anthropology, which position medicines as commodities with life stages, playing various roles in each stage to restore, improve and maintain health. The stage of medication death reflects the consumption and administration of medicines, with the afterlife where the desirable effects of medicines are produced within the human body. According to this concept, wasting and destroying medicines that are unused and unexpired, and not reusing them, results in a meaningless existence for the medicines themselves because they are not used to their complete potential. Thus, many regretted that medicines were being “wasted” and especially as it looked like “nothing was wrong” with them, where the outer appearance remained intact. For example:

“*Please do it. I have had to return medication in the past just for it to be thrown away. It is wrong and wasteful when there is nothing wrong with it*.”

(Participant 112).

“*I think it is a brilliant idea. I have returned medication to the pharmacy in the past and thought it wasteful to destroy*.”

(Participant 135).

“*Having had to return medication from 2 people who died and had much surplus, it has always seemed to be to be such a waste*.”

(Participant 177).

#### 3.1.3. Social and Economic Benefit

Here, people see medicines as commercial goods. The exchange of medicines allows people to meet their health requirements, and businesses to meet their targets and profits. Within this theme, reusing medicines, i.e., the re-exchange of pharmaceuticals between pharmacies and patients, benefits the public and the NHS by reducing healthcare costs and medicinal waste. This was the most commonly occurring theme. Many individuals positively encouraged the reuse of medicines because, they postulated, this would help the economy, i.e., reduce NHS and patient expenses, reduce waste produced from the destruction of unused and unwanted medicines, and allow the environment to be kept cleaner by minimizing landfill waste. In general, patients expressed a clear link between reusing medicines and a reduction in healthcare costs. This suggests medicines are given an economic value, similar to other common commodities. For example:

“*I am entirely in favour of reusing medication. Far too much is wasted at great expense to the NHS and thus the taxpayer*.”

(Participant 10).

“*Blisters go to landfill and cannot be recycled*.”

(Participant 40).

“*I believe that unused, unopened pills should be reused, instead of being destroyed. Even given free to places where medications are too expensive for people who are living in poverty*.”

(Participant 191).

#### 3.1.4. False Analogy

This theme draws upon people’s current knowledge of the types of products that are currently reused within healthcare. The fallacy assumes that if two things are alike in one or more aspects, then they will also be alike in another aspect. Thus, because all pharmaceutical goods including medicines, appliances and devices are used with the intention to diagnose, treat or prevent diseases, reusing one product should mean that all others are also suitable for reuse. For example, if dressings and medical devices that have not been opened or tampered with can be reused, then so can medicines, including solid and liquid dosage forms. For example:

“(reuse) *Applies to other things within NHS e.g., dressings, stoma products*.”

(Participant 21).

“*As long as medication/dressing etc. has not been tampered with, use and not waste them*.”

(Participant 149).

### 3.2. Medicines as Powerful Potions

The four themes within this category relate to the special features of medication that set them apart from ordinary commodities. Less than a quarter of the comments reflected these themes.

#### 3.2.1. The Drug Development Process

Drug discovery and development processes are time consuming, expensive and complex. There are many stages involved in producing highly stable and effective formulations of drugs, including pharmacological and pharmacokinetic testing, along with their manufacturing. Thus, the development and maintenance of medicines being complex, sets them apart from everyday commodities. A limited number of comments reflected this theme. Participants mentioned the need for scientific data, evidence, and published trials to evidence continued drug stability before proceeding with medicines reuse. Some thought that not all types of formulations would be suitable for reuse.

“*Need to see published trials*.”

(Participant 192).

“*Only reuse quality medications not generics*.”

(Participant 88).

“*Adhesive on morphine patches not of best quality*.”

(Participant 82).

#### 3.2.2. Specially Regulated Products

This theme acknowledges the regulations of medicines by authorities such as the medicines and healthcare products regulatory agency (MHRA) to ensure quality, safety and efficacy standards are achieved and maintained before and after the licensing and marketing of medicines. This includes giving expiry dates and specifying storage conditions to preserve and maintain shelf life and prevent drug degradation. Although many of the participants expressed pro-medicines reuse intentions, some still commented on the potential impact of the storage environment on medicines and whether this would affect their quality. Concerns were also expressed on the safety and authenticity of drugs, as it is difficult to verify how and where medicines have been kept and handled. For example:

“*Conditions under which it may have been stored are unknown e.g., insulin in fridge*.”

(Participant 90).

“*Many people will be afraid that re-using meds runs a risk of contamination*.”

(Participant 36).

“*Even though the medication would appear to be sealed in its original packaging you don’t know how it has been stored, this could have an effect on it if stored in too hot or too cold temperatures*.”

(Participant 66).

“*Proof of stability is a big concern*.”

(Participant 38).

#### 3.2.3. Unique to an Individual’s Health

This theme relates to the purposeful selection and prescribing of medicines to treat someone’s health condition. Healthcare professionals will have carefully chosen a specific medicine, from a range of treatment options, to suit the individual’s needs. The therapeutic effects and outcomes of a medicine, it follows, will be dependent on the patient’s unique set of circumstances, as determined by the health professional. Accordingly, medicines should not be shared because their outcomes cannot be guaranteed under a different set of circumstances—instead, when no longer needed, medicines ought to be returned to the pharmacy for disposal. In this way, medicines are quite unlike ordinary commodities. The survey was completed by people with chronic health conditions. Many responded considering their own medicines, such as antidiabetics on which they rely to remain well. In addition, some comments conveyed a strong desire to adhere to advice given by health professionals, as the experts in their field. This theme highlights the complexity of medicines and the supervision that is needed alongside their usage. For example:

“*I am type 1 diabetic and don’t feel that reusing medication is for any diabetic*.”

(Participant 203).

“*I would reuse sealed medication only if my Dr said it was safe*.”

(Participant 5).

#### 3.2.4. Handling to Meet Legal and Practice Guidelines

According to this theme, the dispensing of prescription-only medicines (POMs) carried out by trained staff and checks by pharmacists must be accurate and follow protocols, such as standard operating procedures (SOPs) to maximize patient safety and care. This is because medicines are powerful and valuable and can be susceptible to misuse or cause harm if mishandled. This was a commonly occurring theme. Individuals cautioned against reusing medicines, highlighting negative repercussions if they are handled casually. Thus, quality checks by trained health professionals were deemed essential to assess the safety and appropriateness of medicines for use, central to patient care. A few participants also commented on the possibility of fake medicines entering the supply chain, which further necessitated the need for thorough checks. Thus, medicines are not the same as other commodities as there is a lot more at stake should they be mismanaged.

“*I worry about fake medication*.”

(Participant 28).

“*Providing everything has been checked out by professionals and have long use by date*.”

(Participant 96).

“*There would need to be very strict guidelines in place to ensure patient safety*.”

(Participant 130).

## 4. Discussion

This research is important because it unearths how people think about medicines and the properties that make them reusable or not. The category of “medicines as common commodities” encapsulates commonly held ideas about what makes medicines the same as any other commodity and therefore suitable for reuse, and the category of “medicines as powerful potions” describes what confers medicines their potency and special status, distinct from ordinary commodities thus cautioning against their reuse. These categories highlight the contradictory ways in which medicines can be viewed by different, and sometimes even the same people, and helps explain how medicines can be deemed both reusable and not reusable. The findings can help policy makers understand what makes people (even themselves) receptive to the idea of medicines reuse and importantly, how existing medicines reuse practices (e.g., visual inspections) might in fact be more in line with the everyday view of medicines as common commodities rather than the “powerful potions” view normally advocated by biomedicine.

A strength of this study is that the primary data came from a survey that captured views about medicines reuse from a representative sample of the UK patient population [22]. The sample was representative in terms of gender, ethnicity, geographical location and education level. As such, the data can be generalised to the wider population, and therefore displays some level of external validity. Additionally, as the data came from patients with chronic conditions who are more likely to be using medicines regularly, their opinions towards reuse would be expected to be more meaningful than if gathered from healthy volunteers. A weakness is that the study relied on the analysis of static comments written in an online survey where it was not possible to seek further information or justification to the answers provided. Another limitation of the study is that, although sufficient for a qualitative analysis, only 210 respondents made written comments on the questionnaire, which represented a fifth of the overall number of participants.

The majority of the comments from the survey belonged in the category of medicines as common commodities, with only a quarter reflecting medicines as powerful potions. Commodities are standardized goods or services enabling an exchange or sale between the manufacturers/providers and consumers; of economic value, commodities are primarily produced to meet market demand and satisfy individuals’ needs [24]. Medical anthropologists’ examination of medicines as commodities positions these with social, cultural and economic aspects far beyond their material (chemical) properties [25]. As such, medicines are commodities for exchange with social lives, and different life stages and roles as they move from one setting to another, i.e., from manufacturers to marketing, prescription by healthcare professionals, and dispensing by pharmacies for patients’ use [26]. This notion of commodification contrasts with a biomedical understanding, where, in line with the category of powerful potions, medicines are classified according to potency, are restricted and regulated in their use and given specific directions for storage and administration, marking them as highly distinct from everyday commodities.

Dichotomous representations of medicines are not new. For example, in previous work, medication has been described as “marvellous medicines” versus “dangerous drugs” [27]. Similarly, when antipsychotics are prescribed in dementia, they are either “the lesser of two evils” or “medicines not smarties” [28]. In this study, the dichotomization explains what on the one hand permits the reuse of medicines but on the other inhibits it. The participants’ notion of medicines as ordinary commodities was most commonly captured by the theme of social and economic benefits, followed by the themes of the social life of medicines and physical appearance. Thinking about medicines in terms of their economic value is not new and examining the literature, studies promoting medicines reuse, including our own [20], do tend to cite cost savings as a viable reason to explore the practice [29,30]. However, the economic argument is only part of the equation.

In their 1989 paper, Van der Geest and Whyte [31] argued that the “thinginess” of medicines makes them democratic; medicines are thought to contain the power of healing in themselves, thus anyone who “gains access to them can apply their power”. This is what makes medicines transactable and subject to commoditization. However, Van der Geest and Whyte [31] also argued medicines are “enclaved commodities” because their biochemical properties necessitate in-depth knowledge about disease and people’s functioning when they are handled; health professionals thus attempt to limit access to medicines to prevent waste, misuse or harm. This is despite countless strategies of diversion by people that include selling, stealing, smuggling, hoarding, forging, exchanging and using medicines as gifts [31]. Seen in this light, it could be argued that the category of medicines as “powerful potions” is in line with health professionals’ view of them as “enclaved commodities”, which explains why returned medicines are normally kept safe by pharmacists and sent away for disposal (so that further access to them is prevented). Indeed, the need to meet legal and practice guidelines, and being specially regulated products, were the main themes that distinguished medicines as powerful potions that needed special caution if to be reused. On the other hand, it could be argued that seeing medicines as “common commodities”, in line with the notion of the democratization of medicines explains why so many patients returning their unwanted medicines to pharmacies voice a request for these to be reused. It also explains the illicit medicines reuse practices identified in the introduction to this paper.

Seeing medicines as both similar and different to other commodities at the same time is perhaps the key to why even government decision makers are willing to accept the notion of medicines reuse under certain circumstances even when this practice is normally unthinkable to them. For example, medicines reuse was permitted when the availability of medicines was threatened during the recent pandemic [17]—presumably because it was better to have a product available, albeit one that might be less potent, to meet market demand, than to have none at all. However, the dichotomization unearthed in this study does not actually justify this approach. After all, as argued earlier, the visual inspection of medicines cannot actually guarantee their safety. This is because it is possible that the active ingredient of the medication degrades, or the formulation breaks down so that ultimately less of the medicine is available to treat the disease, even if the packaging passes visual checks. Due to the plausible weaknesses in mere visual checks, we propose a more robust mechanism using the novel ReMINDS (www.reading.ac.uk/ReMINDS; accessed on 16 April 2021) ecosystem as a solution for reusing returned prescribed medicines. This system relies on active sensing technologies integrated with the Internet of Things platform to validate the quality and safety of the medicines while interconnecting the relevant stakeholders [32,33]. Such a system would acknowledge medicines as both “powerful potions” but also as transactable things, subject to commoditization. In this way, it would be possible to recognize the social and economic benefit of medicines reuse without relinquishing the biomedical principles that ensure the potency and special status of medicines. Future studies will aim to explore the use of such technologies in order to make medicines reuse a safe and effective process.

## 5. Conclusions

This study unearthed people’s interpretations of the properties of medicines that make them reusable or not reusable. Two categories of “medicines as common commodities” and “medicines as powerful potions” were generated. Although these categories appear to contradict each other, they conceivably also provide the key as to why people want medicines reuse to take place on a wider scale and why even governments allow the practice in emergencies. Arguably, even health professionals and policy makers who advocate medicines reuse based on cursory visual checks are won over by the argument of medicines as common commodities in contrast with their biomedical training which normally safely posits medicines within the realm of “powerful potions”. However, rather than compromising on quality and safety in order to meet market demands, developing and using active sensing technologies could be the key to ensuring a plausible medicines reuse practice in the future, allowing the value and social life of medicines to be fully realized while protecting the public from potential harm.

## Figures and Tables

**Table 1:** The concepts developed after the analysis of medicines reuse beliefs, including the two main categories “medicines as common commodities” and “medicines as powerful potions” with their themes and explanations.

Medicines as Common Commodities	Medicines as Powerful Potions
This category encapsulates commonly held ideas about what makes medicines the same as any other commodity and therefore suitable for reuse.	This category describes what confers medicines their potency and special status distinct from ordinary commodities, thus cautioning against reuse.
*Physical appearance*	*The drug development process*
The external features and overall physical appearance of a commodity are adequate to indicate what is held within. Therefore, intact sealed packaging of medicines suggests an authentic product of good quality inside.	Drug discovery and development processes are time consuming, expensive and intricate. Numerous stages ensure stable and effective final formulations, making medicines complex compared to other commodities.
*Social life of medicines*	*Specially regulated products*
Medicines have metaphorical life stages, with a medicine’s death (when consumed) resulting in its afterlife (internal effects) to restore, improve or maintain health. Failure to reuse unused medication therefore makes its existence meaningless.	Medicines are strictly regulated by authorities to illustrate quality, safety and efficacy before and after authorization. This includes giving expiry dates and storage conditions to maintain the shelf life.
*Social and economic benefit*	*Unique to an individual’s health*
Here, medicines are standardized commercial goods with economic value, exchanged between manufacturers and consumers to meet their needs. Reusing medicines thus brings benefit by reducing medicines spending and waste.	Medicines are prescribed for specific individuals with the unique therapeutic effects dependent on the individual’s circumstances. Medicines must not be reshared as their outcome in others cannot be guaranteed.
*False analogy*	*Handling to meet legal and practice guidelines*
This fallacy assumes that if two things are alike in one aspect, then they will be similar in another aspect too. Thus, if devices and appliances used to diagnose and treat health conditions can be reused, then so can medicines.	The sale or exchange of medicines (over the counter or via prescription) must adhere to legal protocols and accuracy and clinical checks. As powerful substances, their casual handling could cause harm to patients.

## Data Availability

The authors can be emailed for further information about the data.
